# Pharmacological manipulation of the cell cycle and metabolism to protect normal tissues against conventional anticancer drugs

**DOI:** 10.18632/oncotarget.265

**Published:** 2011-04-20

**Authors:** Ingeborg M.M. van Leeuwen, Sonia Laín

**Affiliations:** ^1^ Microbiology, Tumor and Cell Biology, Karolinska Institutet, Nobels väg 16, 171 77 Stockholm, Sweden; ^2^ Centre for Oncology and Molecular Medicine, University of Dundee, Ninewells Hospital and Medical School, Dundee DD1 9SY, Scotland, UK

There is no novelty in stating that conventional cancer therapeutics are highly mutagenic, unselective and toxic. Hair loss, neutropenia, vomiting, and immune suppression are just some of the immediate side effects. In addition, genomic damage induced by these agents can result in second tumours later in life. Cancer cells differ from normal cells in a number of hallmarks that define the tumour phenotype [1]. These traits, which involve genetic and epigenetic changes in tumour suppressors and oncogenes, constitute a double-edged sword for cancer cells. They provide a proliferative advantage but, importantly from a treatment prospective, also mean an increased dependence on certain cellular functions as well as an increased vulnerability to particular insults. The goal of cancer treatment is to exploit this differential sensitivity to selectively kill tumour cells whilst sparing normal tissues. Classically, the search for new anticancer therapies has focused on the discovery of agents that annihilate tumour cells at least as effectively as existing chemotherapeutics whilst having weaker or negligible effects on healthy tissues. Alternatively, so-called *cyclotherapy strategies* aim at improving the therapeutic window by selectively shielding normal cells from conventional anticancer drugs [2,3]. The majority of such drugs target dividing cells. Hence, if we could transiently and selectively pause proliferation in normal tissues, subsequent treatment with S- or M-phase poisons should eradicate cycling tumour cells only.

Nutlin-like compounds are well-established non-genotoxic activators of the p53 pathway [4] currently undergoing clinical trials for treating patients with cancers retaining wild-type p53. In addition, nutlin-3 has been proposed as a potential chemoprotective agent for patients bearing tumours with mutant p53. The rationale behind this use is that nutlin-3 has been shown to have a mild, reversible cytostatic effect on a variety of normal cells [5,6]. We believe it is important to highlight these observations, as a widely spread view is that activating p53 *in vivo* would trigger severe cytotoxic responses in normal tissues. Experimental data supporting this notion comes from animal studies where the expression of p53's major negative regulator, mdm2, was suppressed [7]. However, such undesired side effects do not occur when p53 is activated in a buffered manner using nutlin-3, an agent that binds directly to mdm2 [4], partially impairs mdm2's down-regulating activity on p53 [8], and stabilises mdm2 [9]. Indeed, nutlin-3 has been shown to protect normal cells from mitotic poisons such as the aurora kinase inhibitor VX680 [10], taxanes [5,6], and polo-like kinase inhibitors [11]. Most interestingly, nutlin-3 protects mice from PLK1 (polo-like kinase 1) inhibitor–induced neutropenia without abating the anticancer potency of the mitotic poison [11]. In their recent article, Apontes *et al.*, (2011) provide additional evidence supporting the potential suitability of nutlin-3 for cyclotherapy purposes. Indeed, nutlin-3 also protects normal cells from nocodazole, a compound that –like the vinca alkaloids used in the clinic– inhibits tubulin polymerisation.

**Figure 1:**
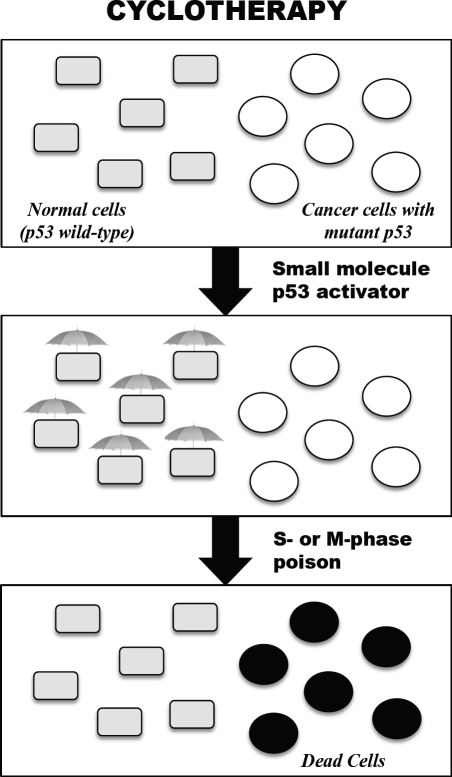
Cartoon illustrating the concept of p53-based cyclotherapy The p53 tumour suppressor is mutated in about 50% of human solid tumours in adults. Hence, administering a small-molecule p53 activator to patients bearing p53-mutant tumours would selectively activate p53 in normal tissues, inducing a mild reversible cell-cycle arrest. Subsequent treatment with conventional anticancer drugs should then kill proliferating cancer cells, whilst leaving normal cells untouched.

The results summarised above suggest that nutlin-3 could constitute an ideal agent for chemoprotection purposes in patients with p53-deficient tumours. However, the clinical use of this compound has not yet been approved. In a recent paper, we tested the possibility of using low doses of actinomycin-D, an approved drug that at nanomolar concentrations can increase p53 activity in a similar manner to nutlin-3 [12]. Yet, there are some important differences between the p53 responses to nutlin-3 and LDActD that can be understood on the basis of their different from mechanisms of action [9]. Like nutlin-3, LDActD has a primarily cytostatic effect on p53 wild-type normal cells and protects them from subsequent treatment with VX680 [13]. Unfortunately, LDactD pretreament also protects p53-deficient tumour cells against VX680 to a small extent. Our hope is that LDActD might perform better in a p53-based cyclotherapy setting when combined with S-phase poisons than with mitotic poisons. In this line, nutlin-3 has been shown to prevent the mutagenic and cytotoxic effects of gemcitabine and cytosine arabinoside in normal cells without diminishing the killing effect of these compounds on p53-mutant tumour cells [14].

In the article featured here [6], aside from nutlin-3, Blagosklonny's group evaluate other clinically-approved drugs as chemoprotective agents against the tubulin poisons nocodazole and paclitaxel. The two well-known drugs chosen for this purpose are rapamycin, a drug with striking anti-ageing properties, clinically used to prevent transplant organ rejection (in chronic administration with traditional immunosuppressants) [15], and the anti-diabetic drug metformin. The authors show that pretreatment with these drugs succeeds in protecting all normal cell types tested against these mitotic poisons, whilst having little impact on the vulnerability of a cancer cell line with mutant p53. The chemoprotective effects might be related to the interesting observations that glucose starvation selectively protects primary cells against cyclophosphamide in cell culture and fasting reduces the side effects of chemotherapy in patients [16,17]. Furthermore, evidence suggests that lack of p53 may sensitise cancer cells to metabolic stresses such as nutrient deprivation [18]. The study by Apontes *et al.* also addresses whether combining two chemoprotective agents could lead to an even greater increase in the therapeutic window of mitotic poisons. In this regard, nutlin-3 plus rapamycin and rapamycin plus metformin gave the most promising results.

Like in the case of the nutlins, the efficacy of metformin and rapamycin is currently being assessed in clinical trials for cancer [19,20]. Independently of whether these tests are successful, the encouraging data presented by Apontes and co-workers suggests that there might be other avenues for these compounds in cancer therapy.
